# Examining Gender Differences, Personality Traits, Academic Performance, and Motivation in Ukrainian and Polish Students of Physical Education: A Cross-Cultural Study

**DOI:** 10.3390/ijerph17165729

**Published:** 2020-08-07

**Authors:** Cezary Kuśnierz, Aleksandra M. Rogowska, Iuliia Pavlova

**Affiliations:** 1 Faculty of Physical Education and Physiotherapy, Opole University of Technology, 45-758 Opole, Poland; c.kusnierz@po.edu.pl; 2 Institute of Psychology, University of Opole, 45-052 Opole, Poland; 3 Theory and Methods of Physical Culture Department, Lviv State University of Physical Culture, 79007 Lviv, Ukraine; pavlova.j.o@gmail.com

**Keywords:** academic motivation, academic achievement, Big Five personality, gender differences, cross-cultural differences

## Abstract

Background: This study examined the relationship of academic performance with the Big Five traits of personality, academic motivation, and gender in a cross-cultural context. Methods: Participants in the study were 424 university students of physical education (PE) departments from Poland (53%) and Ukraine (47%). Undergraduates completed a brief version of the International Personality Item Pool (Mini-IPIP) to assess the Five-Factor model of personality, the Academic Motivations Scale (AMS), and grade point average (GPA). Results: Polish PE students scored higher in emotional stability and extroversion and had a higher GPA than Ukrainian PE undergraduates. Gender differences were found in both personality traits and academic motivation scales. Intrinsic motivation may predict academic performance. Conscientiousness and intellect emerged as mediators of the relationship between intrinsic motivation and academic performance and gender was found as a moderator in the relationship between conscientiousness and academic success. Conclusions: Women are more motivated regarding academic achievements than men. In addition to intrinsic motivation, the most important factors for academic grades are some personality traits, gender, and cultural differences. Openness and conscientiousness in men are mediators between intrinsic motivation and academic performance. The results of this study may be useful for PE academic teachers to improve the motivation of their students.

## 1. Introduction

There is significant evidence that regular physical activity (PA) has beneficial effects on the physical, mental, and social aspects of well-being and contributes to the academic achievement of children and adolescents, e.g., [1,2,3,4]. The habit of being physically active during childhood increases the chances of leading a healthy lifestyle for one’s lifespan [3,5,6]. School is the best environment to facilitate healthy habits and physical education (PE) classes are the best way to develop PA. Physical education plays a crucial role in the acquisition of competencies that contribute to motor, cognitive, personal, emotional, and social development [7].

PE teachers play a key role in promoting and modeling health-related behaviors, in transforming students’ attitudes and beliefs about learning, and developing intrinsic motivation and personal growth [6,8,9,10,11]. In particular, research indicates that PE teachers have a real influence on motivation and the intention to be physically active among their students [11]. A review study found that self-determined motivation is positively related to various affective, cognitive, and behavioral outcomes [12]. Thus, Orsini et al. [12] postulate that autonomic motivation to engage in academic activities can be enhanced in health education by an early detection of students’ characteristics, as well as by changes in the educational environment. Autonomous motivation can predict an individual’s engagement and performance in both competitive sport and recreational physical activity [6,13,14,15]. Moreover, motivation can explain participation in and withdrawal from physical exercise during leisure time, in addition to participation in school-based physical education lessons [6,13,16]. However, a deeper understanding of why people participate in PA should take into account a wide range of variables, including personality, cultural, and gender differences [13].

The present study aimed to examine cross-cultural differences in academic achievement, autonomous motivation, and personality among Polish and Ukrainian students of PE departments. There are several reasons for studying the above variables in future teachers of PE. According to the concept of sustainable academic motivation, the explanation of student motivation should be considered in relation to teacher motivation [17]. Indeed, recent research showed that the perceived transformational relationship between PE teachers’ behavior and students’ PA was stronger when students were harmoniously passionate and self-determined [8]. Furthermore, Abós et al. [18] showed that autonomously motivated PE teachers, who enjoy and value their teaching, reported lower levels of emotional exhaustion. Thus, autonomous motivation may increase job satisfaction and mental well-being and may also prevent burnout among PE teachers. In contrast, the phenomena of high stress and school withdrawal among beginning physical education teachers has become a major problem in education systems in recent decades; approximately 50% of all new teachers in the United States and Europe leave the profession within the first five years [19]. Among the many barriers, one of the most frequent issues reported by the vast majority of novice teachers is an insufficient level of preparation for the realities of the teaching profession. Therefore, academic achievements and their determinants in PE university students should be the subject of systematic research.

### 1.1. The Relationship between Academic Performance, Personality, and Motivation

Motivation is fundamental to human behavior in both adaptive and maladaptive dimensions. Motivation is determined by a complex interplay of internal and external factors, including individual differences in expectations, self-efficacy, self-regulation, goals, task values, and perceived costs and benefits [20]. According to self-determination theory (SDT), motivation is considered to lie on a continuum, ranging from intrinsic motivation through extrinsic motivation to amotivation [21,22,23]. Students with intrinsic motivation (IM) demonstrate a high level of internal locus of control and enjoyment of learning. People with high IM tend to seek cognitive stimulation, challenge, and competition; are enthusiastic; engage in an activity for the associated pleasant sensations and satisfaction related to exploration; and are ready for long-term effort to improve skills and achievements. Extrinsic motivation (EM) is related to the achievement of contingent goals. It is regulated externally under the influence of the environment and its constraints, rewards, and punishments by internal pressures (the pursuit of pride or avoidance of guilt and shame) or identification with a personally endorsed value or objective. The third primary dimension of SDT is amotivation (AM), a lack of student motivation or intention toward the target behavior. Individuals with high amotivation do not respond to environmental influences, have reduced achievement needs, and show a tendency to disengage or drop out rather than task resolving, attending classes, and studying. Intrinsic motivation and identified regulation (as a type of well-internalized external motivation) are considered autonomous forms of behavioral regulation.

Consistent with the SDT assumptions, autonomous motivation may be enhanced by support for students’ basic psychological needs for autonomy, competence, and relatedness [22]. Autonomous motivation was found to predict more positive outcomes across varied educational levels and cultural contexts than externally controlled forms of motivation and amotivation [22,24]. Various studies also consistently showed a positive relationship between intrinsic motivation and academic achievement [22,24,25]. There is also some evidence that intrinsic motivation is associated negatively with amotivation and reciprocally determines school achievement [25]. A meta-analysis found that 16.6% of the variance in school achievement was uniquely explained by motivation [22,24,25,26].

Personality may be considered a theoretical construct aimed at describing, explaining, and predicting the way human beings function in various aspects of life. Of the various psychological paradigms, the Big Five model of personality is the most frequently used to assess dimensions such as neuroticism (emotional stability), extroversion, agreeableness, conscientiousness, and openness to experience (intellect) [27,28]. The personality traits are determined biologically and environmentally. Komarraju et al. [29,30,31,32,33,34,35,36,37] showed that Big-Five personality factors contribute to academic success, including exam performance and grade point average (GPA). Research indicates that of the five personality traits, only conscientiousness was consistently the strongest predictor of academic performance, whereas the other four traits have weak or mixed relationships with GPA [35,36,37]. In particular, academic success (GPA) is related to a proactive aspect of conscientiousness, such as being hard-working and persistent [30]. A systematic review and meta-analysis of the relationships between the Big Five personality traits and tertiary academic performance indicate that agreeableness and openness were also positively correlated with GPA, in addition to conscientiousness [36]. Furthermore, the academic major was found as a moderator in the relationship between conscientiousness and GPA.

Personality traits also distinguish athletes and physically active individuals from the population characterized by a more sedentary lifestyle. Research indicates that a high level of extroversion and conscientiousness and low levels of neuroticism contributes to physical activity [38]. Moreover, openness seems a significant predictor of moderate physical activity in females, whereas agreeableness, emotional stability, and conscientiousness were associated with vigorous physical activity in males [39]. However, recent research did not find significant differences in Big Five personality traits between university students of physical education and other academic majors [40]. In particular, little is known about the relationship between academic performance and the personality of PE students. There is evidence that participation in formal sporting activities at university is associated with higher academic performance (i.e., GPA) among undergraduates [41]. However, the results of a systematic review suggest that only 50% of reviewing publications showed a positive association between physical activity and academic performance [42]. Other studies did not demonstrate a statistically significant relationship between these two variables.

Mcabe et al. [43] showed that conscientiousness was strongly and positively related with mastery-approach goals, characterized by frequently used task-referenced and self-referenced competence standards. This type of achievement motivation is more like intrinsic motivation. Some studies found an association between Big Five personality traits, motivation, and academic performance [29,44,45]. The highest academic achievement (first-quarter GPA) was related to high levels of conscientiousness and intrinsic motivation and low levels of extrinsic motivation, among non-traditional undergraduates at a Hispanic-serving institution [44]. Another study indicated that higher annual academic performance was demonstrated by the most conscientious and intrinsically motivated French management students [45]. Conscientiousness was found to be the strongest predictor of GPA and a moderator between amotivation and academic achievement.

Komarraju et al. [29] examined the interrelationship between GPA, personality, and motivation. IM was predicted by conscientiousness and openness, EM by conscientiousness and extroversion, and AM by conscientiousness and agreeableness. All personality traits, except extroversion, were also related to self-reported GPA (and explained 14% of the variance in GPA). Three scales of motivation—IM, EM, and AM—explain collectively 6% of the variance in GPA. However, only intrinsic motivation was a significant predictor of academic performance, explaining 4% of the variance in self-reported GPA. The relationship between intrinsic motivation to accomplish and GPA was partially mediated by conscientiousness.

There is some evidence that conscientiousness interacted with self-motivation to predict university GPA [46]. More specifically, conscientiousness and self-motivation compensated in predicting the GPA of college undergraduates. Better academic performance demonstrated highly conscientious students or those high in self-motivation. This compensatory function of conscientiousness was confirmed in other studies since conscientiousness was a stronger predictor of GPA at lower levels of autonomous motivation [47]. Similarly, Zhou [48] showed that conscientiousness and agreeableness positively predicted academic performance when self-determined motivation was low in primary school students in China.

### 1.2. Gender Differences in Academic Performance, Motivation, and Personality

Numerous studies indicate gender differences in school achievements, motivation, and personality. Although both genders demonstrate the same general level of intellectual ability, females are reported to outperform males in academic achievement at different stages in the school system, having better grades and reaching post-school qualifications in higher numbers [49,50,51,52].

Studies indicate that female students scored higher than males in internal and external motivation but have lower values in amotivation [23,53,54,55]. Furthermore, among Polish students of such academic majors as physical education, physiotherapy, tourism, recreation, sport, and sport and tourism management, females scored higher than males on the following scales of the Academic Motivations Scale (AMS): external regulation, introjected regulation, identified regulation, and internal motivation to know [56]. A higher score in amotivation was reported in males compared to female students. Statistically, significant gender differences were not noted in two scales of internal motivation: to accomplish and to experience. There is also evidence that motivation for engaging in a physical activity differentiated in people dependent on gender [57]. Male students are more motivated by intrinsic factors (like the need for power, competition, and challenge), whereas extrinsic motives dominate among females (e.g., body weight control and appearance).

Gender differences in personality were found in many studies but results of the research were inconsistent [58,59,60,61,62]. For example, Mac Giolla and Kajonius [59] showed that women scored higher than men on all of the Big Five traits of personality and that these differences were larger in more gender-equal countries. However, in other studies, no gender differences were found in openness [58,60,61,62], conscientiousness [58,62], or extroversion [61]. Thus, more research is needed to explain mechanisms of gender differences and disparities in particular previous research.

### 1.3. Cross-Cultural Differences

Culture refers to the integrated pattern of human knowledge, history, tradition, language, and belief systems that affect the perception of the world. Culture is a configuration of thoughts, feelings, and behaviors shared by group members as an effect of learning and transmitting knowledge to succeeding generations [63]. Previous research found some cross-cultural differences among undergraduates in motivation and academic achievements [43,44]. Ardeńska et al. [64] showed cross-cultural differences in motivation between Polish and Turkish students of PE departments. Turkish PE students (in particular female) were less amotivated and more intrinsically motivated compared to Polish PE students. In addition, several differences were found between Spanish and Romanian university students in their motivation for sport (task- versus ego-oriented) and its association with family functionality and level of PA [65]. Arvidsson [66] investigated motivation in a sample of Asian and non-Asian undergraduates in the USA and 20 students from Hong Kong. Asian undergraduates showed a higher level of amotivation and lower intrinsic motivation compared to non-Asian students, although both samples did not differ in terms of GPA. Comparing Asian and Western cultures, Boyle et al. [43] found that Asian undergraduates have higher GPAs than non-Asian students in Australia. In addition to goal orientation (both approach and avoidance), country was a significant predictor of GPA, revealing a cultural influence on academic performance among university students. Although the universality of the structure of the Big Five model of personality was confirmed in numerous studies, cultural differences were found in trait levels and gender differences [67,68,69]. However, little is known about cross-cultural differences in the association between motivation, personality, and academic performance in university students.

In particular, there is sparse knowledge regarding the differences between Polish and Ukrainian PE students. Each year, between 6000 and 9000 Ukrainian students go to Poland to study various majors at Polish universities. Currently, more than 35,000 Ukrainians study in Poland. Most of these are from the western regions of Ukraine. According to the latest data, 55% of foreign students in Poland are Ukrainians, indicating that a deeper understanding of the motivation of Ukrainian students to study is required. Previous research found underperformance among ethnic minority students in general education, including in areas of study such as knowledge and skills assessments [70]. Differences in motivation were also found between ethnic minorities and the majority of PE students, but the results were ambiguous and need further study.

In Poland, compared to Ukraine, the system of admission to higher education is easier. The reason for their popularity among Ukrainian students is that Polish universities are constantly improving the curricula for foreign students, providing new opportunities. Ukrainians are also attracted by the opportunity to study in both Polish and English. In addition, the Polish education system guarantees a European-style diploma, which has significantly more prospects. Some Polish universities, accredited by British or North American universities, provide the opportunity to obtain not only a diploma from their educational institution, but also a diploma from one of the universities of these countries. This greatly expands the opportunities for master’s studies to include other developed countries. This means that migration activity related to education between Poland and Ukraine will continue to grow.

The cultural closeness of peoples should also be taken into account. Poland and Ukraine have a large number of common characteristics, including geographical territory (bordering countries), history (a significant portion of the current West Ukraine previously belonged to Poland), religion (predominantly Catholic), and similar political and socio-economic context related to the influence of the Soviet Union in the previous century. However, for the past 16 years, Poland has been a member of European Union (EU), whereas Ukraine is a candidate to join. This determines educational, socio-cultural, and economic differences (Ukraine is a lower-middle-income country, whereas Poland is classified as high income). The most recent reforms of the education systems in Poland and Ukraine relied on adapting them to the standards of the EU. In recent years, PE programs in the USA and EU have started to take greater account of the needs of children and young people, in addition to the development of personal and social skills [7]. From this perspective, it is of interest how these changes in PE distinguish the two countries: Poland and Ukraine. In particular, various aspects related to learning and motivation in Poland and Ukraine remain unexplored.

### 1.4. Current Study

Knowledge about the academic achievements, motivation, and personality of PE students will allow study programs to be better adjusted and the required professional skills and competencies to be acquired in line with expectations and predispositions. Previous studies indicate that academic achievement depends on individual differences in motivation and personality [25,29]. However, little is known about gender differences in the association of academic performance with personality and academic motivation. Vedel et al. [36] showed that an academic major might contribute to the association between personality traits and academic performance. However, most of the previous research was conducted among psychology students, which cannot be generalized to an entire academic population. In particular, three variables, namely, motivation, personality, and academic performance, have not been tested collectively in a sample of physical education students in a cross-cultural context. Thus, for the first time, this study examines the relationship between the Big Five traits, academic motivation, academic performance, and gender in Polish and Ukrainian samples of physical education (PE) students.

The following research questions were examined: (1) Are there gender differences in personality traits, academic performance, and motivation? (2) Do Polish students of physical education differ from Ukrainian PE students in personality traits, academic performance, and motivation? (3) What is the relationship between academic performance and personality and academic motivation? Based on the previous research mentioned above, we hypothesize that: (1) Women score more highly than men in academic achievements, personality traits, and intrinsic and extrinsic motivation. (2) Some cross-cultural differences are expected in GPA, motivation, and personality. (3) Academic performance is related to personality traits and intrinsic motivation.

## 2. Materials and Methods

### 2.1. Participants and Procedure

Participants in the study were 424 university students of PE departments, with men comprising 62% of the total sample (*n* = 161). The age of participants ranged from 18 to 29 years old (*M* = 20.01; *SD* = 2.48). The total sample of PE students consisted of Polish (*n* = 224, 53%) and Ukrainian participants (*n* = 200, 47%). The majority were undergraduates (*n* = 342, 80.66%) in their first year of study (*n* = 220, 51.89%) and with a specialization as an instructor/coach (*n* = 245, 57.78%). Table 1 shows the prevalence of demographic variables by country and gender.

During each semester of study, Polish PE students complete 25 academic hours weekly (1 academic hour = 45 min), 40% of which are practical training, 30% module pedagogical and psychological, and 30% humanities and foreign languages. Students with a PE major may choose one of two specializations: PE teacher or instructor/coach in selected sports disciplines. PE students have an average of 10 h of PA classes each week, each semester, and each year of study. Consistent with individual PA interests, students can attend swimming, athletics, team sports (football, handball, volleyball, basketball), and individual sports (e.g., fitness, self-defense, aerobics, table tennis, strength exercises). Moreover, PE students must complete one summer camp of 60 h (with swimming, sailing, and windsurfing) and one winter camp (45 h of skiing, downhill skiing, and snowboarding) during the first-degree study. The majority of PE students are members of the Academic Sports Association, so they systematically participate in additional sports training and sports competitions in their leisure time. All Ukrainian PE participants studied at the Lviv State University of Physical Culture, which offers only one specialization in Physical Education and Sport. The Ukraine physical education curriculum is similar to that of Poland. Consistent with this, a “double diploma” can be earned in this field between Polish and Ukrainian universities. In the process of learning, Ukrainian students receive the same competencies as Polish peers and can study the same disciplines in the same number of hours. Differences in study plans are less than 15–20%.

After the bachelor’s degree graduation, graduates do not have any qualifications to teach or coach. Graduation from a master’s in PE provides qualifications to teach PE in primary or secondary schools. In addition, students who graduate with the instructor/coach specialization have permission to be an instructor or coach in selected disciplines. However, these qualifications require the completion of an increased number of classes in activities such as training in selected sports, practicing in sports clubs, completing instructor/coach dissertations, and passing additional exams.

Cross-sectional survey studies were performed concurrently at two large universities, one in Poland and one Ukraine, during the second semester of the academic year 2019/2020. The inclusion criterion was to be a student of a PE major. We did not limit the study sample to other inclusion or exclusion criteria. Students of the PE departments voluntarily and anonymously completed the paper-and-pencil standardized questionnaire in their native language, during classes at university, with the consent of lecturers. A package of questionnaires with questions about demographic data was distributed during classes. All three questionnaires were completed on the same day in one lesson. The average time of completion of the questionnaires was approximately 20–25 min. All subjects gave their informed consent for inclusion before they participated in the study. The study was conducted in accordance with the Declaration of Helsinki and the protocol was approved by the Ethics Committee of Scientific Research at the University of Opole (No. 1/2020, dated 22 April 2020) and the Bioethics Committee of Lviv State University of Physical Culture (No. LSUPC#2019-03-0903, dated 9 March 2019).

### 2.2. Measures

#### 2.2.1. Academic Performance

Undergraduates provided a GPA using a six-point scale (2.0 = Insufficient, 3.0 = Sufficient, 3.5 = Satisfactory, 4.0 = Good, 4.5 = Very good, 5.0 = Excellent), which is consistent with both the Ukrainian and Polish academic grade system. High values indicate the best academic achievement. GPA refers to students’ cumulative grades, calculated by averaging course grades of the previous semester. The participants were requested to obtain their grades from the university’s records and registration office through individual online access. If a participant experienced difficulties accessing their online student account, he or she was asked to provide a probable average assessment. Participants were recruited early in the second semester of the academic year (winter–spring 2020) and provided a GPA from the first semester of the academic year (fall–winter 2019).

#### 2.2.2. Academic Motivation

According to self-determination theory, the Academic Motivations Scale (AMS) is a 28 item measure of academic motivation [23]. Participants rate each item on a seven-point Likert scale (1 = Strongly disagree, 2 = Disagree, 3 = Slightly disagree, 4 = Don’t know, 5 = Slightly agree, 6 = Agree, 7 = Strongly agree). The AMS consists of three scales derived from higher-order factor analysis and seven subscales of lower order (four items per each subscale), including three subscales of Intrinsic Motivation: To Know (IMTK), To Accomplish (IMTA), and To Experience (IMTE); three subscales of Extrinsic Motivation: External Regulation (EMER), Introjected (EMIN), Identification (EMID); and one scale of Amotivation (AM). At a higher level, two types of motivation were also calculated, namely, Extrinsic Motivation (EM, summarized scores from EMER, EMIN, and EMID) and Intrinsic Motivation (IM, consisting of IMTK, IMTA, and IMTE). The Polish validation study conducted with a sample of PE students showed good psychometric properties and the same structure as in the original study [56]. Translation from the English version of the AMS into the Ukrainian language was performed for the present study purpose, according to the standards of cross-cultural adaptation [71,72]. The internal consistency of the motivational scales (Cronbach’s α), as a measure of reliability of the scales, was good in the present study and equaled 0.80, 0.78, 0.79, 0.83, 0.79, 0.74, 0.87, and 0.91.

#### 2.2.3. Big-Five Personality Traits

Donnellan et al. [73] developed the Mini-IPIP as a practically useful and psychometrically acceptable short form of the 50-item questionnaire from the resources of the International Personality Item Pool (IPIP) for measuring the Big Five Factor Model of personality traits. The Mini-IPIP questionnaire consisted of 20-items (both positive and negative), with four items per each of five scales: Emotional Stability (ES), Extroversion (E), Intellect (I), Agreeableness (A), and Conscientiousness (C). Participants reported their resemblance to each item on a five-point Likert scale (from 1 = Not at all accurate, to 5 = Very accurate). The Polish validation study indicated satisfactory psychometric properties [74]. Translation of the Mini-IPIP from the English original version into the Ukrainian language was performed for the present study purpose, according to the standards of cross-cultural adaptation [71,72]. The reliability coefficient (Cronbach’s α) was acceptable in the present study for scales E (α = 0.69), I (α = 0.60), and C (α = 0.67) and poor for A (α = 0.54) and SE (α = 0.57).

### 2.3. Statistical Analysis

The Kolmogorov–Smirnov test was conducted to compare a sample with a reference probability distribution. The distribution of scores in several scales of the AMS and Mini-IPIP were inconsistent with the assumption of normal distribution. However, a deeper analysis of the distribution based on skewness and kurtosis coefficients indicated their sufficient symmetry and similarity to the Gaussian curve because the absolute values of the obtained coefficients did not exceed 1, which indicated good psychometric properties [75]. Furthermore, sample sizes of greater than 85 should be sufficient to generate stable means and standard deviations regardless of the level of skewness [76]. In the present study, the total sample and the particular subsamples divided by gender and country were twice as large as the minimal number. Thus, parametric tests were performed to examine gender and country differences and the association between variables. It is also important to note that most of the statistical tests were reasonably robust in the face of mild to moderate departures from normality and tolerated violations from the normality assumption well [77].

Several statistical tests were performed to answer the research questions and examine hypotheses, including Student’s *t*-test to assess between-group differences, Pearson’s *r* correlation, and hierarchical multiple regression analysis to examine the association between variables. Cohen’s *d* coefficient was used for calculating the effect size and a *p*-value of 0.05 (5%) was adopted as the acceptable level of validity. Academic performance was an explained (dependent) variable in three subsequent models. In the first step, two demographic variables (Gender and Country) were included in the model. The second model also included three motivational scales, IM, EM, and AM, in addition to demographics. The third model consisted of demographic variables, scales of motivation, and personality traits consistent with the Big Five model.

Furthermore, the mediation and moderated mediation analysis were conducted using the PROCESS 3.3. macro for SPSS ver. 25 [78,79,80], with the bootstrap approach to estimate the distribution of the coefficients and the distribution of the prediction errors. A simple mediation and moderated mediation models were performed simultaneously to clarify the relationship between academic performance as a dependent variable and dimensions of motivation and personality as independent variables. Model 4 of the SPSS macro PROCESS [78,79] of mediation and model 14 of moderated mediation were applied for this purpose. The conditional effect was tested based on a bias-corrected bootstrapping procedure with 10,000 samples. Bootstrapping is a nonparametric resampling procedure that involves generating an empirical representation of the sample distribution, treating it as a miniature representation of the population. A bootstrap confidence interval (95% *CI*) that does not include the “0” value signals a significant effect.

## 3. Results

### 3.1. Gender Differences

As shown in Table 2, gender differences were found in both personality traits and academic motivation scales. Women scored higher than men in two scales of IMTK and IMTA and two scales of EMER and EMIR. Female students recorded higher scores than males in IM and EM and scored lower in AM. Women demonstrated higher academic performance (GPA), agreeableness, and conscientiousness and scored lower in emotional stability when compared to men.

### 3.2. Cross-Cultural Differences in GPA, Motivation, and Personality

Table 3 shows the results of the Student’s t-test to examine differences in GPA, motivation, and personality between Polish and Ukrainian PE students. Polish PE students scored higher in emotional stability and extroversion as a personality trait and also had a higher GPA than Ukrainian PE undergraduates. Polish and Ukrainian samples did not differ in academic motivation.

### 3.3. Association of GPA with Academic Motivation and Personality Traits

A correlation analysis revealed numerous significant relationships between GPA and both motivation and personality dimensions. Table 4 presents Pearson’s *r* correlation coefficients between GPA and dimensions of motivation and personality. A positive association was found for academic performance with intrinsic motivation and its two subscales, IMTK and IMTA, in total, Ukrainian, and female samples of PE students. In the Polish sample of PE students, GPA correlated positively with intrinsic motivation and the IMTK and IMTE subscales. Academic performance was unrelated to extrinsic motivation and its three subscales (the EMER, EMIN, EMID) and to amotivation.

Academic performance was positively related to personality traits such as intellect, conscientiousness, and extroversion in the total sample. In Ukrainian PE students, GPA was positively correlated with conscientiousness and intellect and negatively correlated with emotional stability. A positive association was found between GPA and all personality traits except emotional stability in the Polish sample. GPA was positively related to extroversion and intellect in women, whereas it was positively related to conscientiousness and intellect in men.

A hierarchical multiple regression was performed to examine the predictors of academic performance (see Table 5 for more details). The first model of regression (Step 1) included demographic variables such as Gender (coded 0 = Women, 1 = Men) and Country (coded 0 = Ukraine, 1 = Poland). Both female gender and Polish samples of PE students were found to be significant predictors of academic performance. This model explained 8% of the GPA variability, *R*^2^ = 0.08, *R*^2^_adj_ (adjusted *R*^2^) = 0.07, *F* (2, 421) = 17.14, *p* < 0.001. The second model of regression (Step 2) included an additional three scales of motivation: intrinsic, extrinsic, and amotivation. Although internal motivation was a significant predictor, the second model of regression accounted for only 2% additional variance from the first model, *R*^2^ = 0.09, *R*^2^_adj._ = 0.08, *F* (5, 418) = 8.70, *p* < 0.001. Neither extrinsic motivation nor amotivation were predictors of academic assessment. The third regression model for GPA (Step 3) included independent variables such as Gender, Country, three scales of motivation (AM, IM, and EM), and five scales of personality (ES, E, I, A, and C). Statistically significant predictors were Gender, Country, Intellect, and Conscientiousness. The model of regression explained 17% of the GPA variance, *R*^2^ = 0.17, *R*^2^_adj._ = 0.15, *F* (10, 413) = 8.51; *p* < 0.001. Because intrinsic motivation in the third model of regression ceased to be relevant to academic performance, we conclude that personality traits mediate the relationship between these two variables.

According to the results of the hierarchical multiple regression analysis, two mediation analyses were conducted to find the mechanism explaining the academic success. Conscientiousness and intellect were considered as mediators of the relationship between intrinsic motivation and academic performance. The total regression effect of intrinsic motivation on academic performance, ignoring personality traits as a mediator, was poor but statistically significant, *b* = 0.005, *SE* = 0.002, *p* < 0.002. The model of regression explained about 2% of the GPA variance, *R*^2^ = 0.02, *F* (1, 422) = 9.50, *p* < 0.002. The variance increased to 6% after intellect was included in the model of regression, *R*^2^ = 0.06, *F* (2, 421) = 14.12, *p* < 0.001. The direct effect of intrinsic motivation on GPA was slightly lower, *b* = 0.004, *SE* = 0.002, *p* < 0.01. The completely standardized indirect effects of intrinsic motivation on academic performance via intellect was 0.02, *SE* = 0.01, bootstrap *CI* = (0.002, 0.047). Because the bootstrap confidence interval did not include zero, intellect was confirmed as a mediator of the relationship between the IM and GPA.

**Figure 1:**
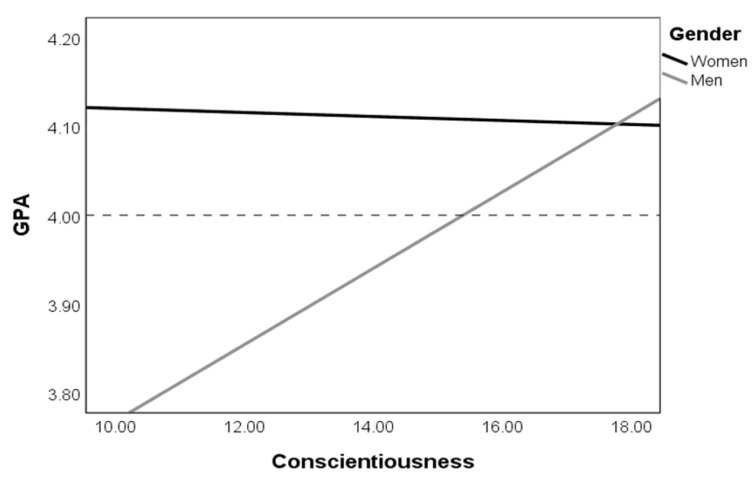
Results of moderated mediation analysis. The dashed line is a mean level of the grade point average (GPA).

When conscientiousness was included as a mediating variable in the relationship between the IM and GPA, the total variance accounted for in the model increased to 5%, *R*^2^ = 0.05, *F* (2, 421) = 10.10, *p* < 0.001. The direct effect of intrinsic motivation on GPA was poorer, *b* = 0.004, *SE* = 0.002, *p* < 0.05. The completely standardized indirect effects of IM on GPA, via C, was 0.04, *SE* = 0.02, bootstrap *CI* = (0.01, 0.07). Because of the 95% confidence interval, which does not include zero, we can assume that the model of mediation was confirmed for conscientiousness as a mediator. Moreover, moderated mediation analysis indicated that gender is a moderator in the relationship between conscientiousness and academic success (Figure 1). Namely, a higher GPA was assigned only to highly conscientious men, whereas women have higher grades independently of conscientiousness level. The moderated mediation model accounted for 9% of the GPA variance, *R*^2^ = 0.09, *F* (4, 419) = 10.37, *p* < 0.001. The indirect effect of the IM on GPA via C was statistically significant only for male PE students, 0.002, *SE* = 0.001, bootstrap *CI* = (0.001, 0.004), whereas for female students, it was not statistically significant, 0.000, *SE* = 0.001, bootstrap *CI* = (−0.001, 0.001).

## 4. Discussion

### 4.1. Gender Differences

Consistent with the hypothesis, this study showed that some personality traits, academic performance, and motivation distinguish women and men studying PE. Female PE students showed a higher GPA than their male counterparts, which may confirm previous studies [49,50,51,52]. In addition, the academic motivation of young adult women in this study was stronger than in men. This was evidenced by higher scores of women in intrinsic and extrinsic motivation scales, in addition to a lower outcome in amotivation compared to men. The results strongly support those of previous studies on gender differences in academic motivation [23,52,53,54,55,56]. Gender differences in academic success and motivation to learn may result from unique patterns of academic and social expectations and social roles for males and females due to the cultural norms in which males are stereotyped as more masculine and assertive than females [49].

Women scored higher than men in agreeableness and conscientiousness and they scored lower in emotional stability (a reverse dimension of neuroticism) compared to men. The present results fully support the findings of South et al. [61] and are partially consistent with other studies [58,59,60,62]. Duckworth et al. [50] showed that girls appeared to be more self-controlled than boys. Costa et al. [68] explain these differences by the attribution of masculine and feminine behaviors to roles rather than traits. A comprehensive genetic study indicates that gender differences in neuroticism, agreeableness, and conscientiousness may be determined by phenotypic variance, but neither genetic nor environmental factors contribute to sex differences in any personality traits [61].

### 4.2. Cross-Cultural Differences

Higher academic performance was found in this study among Polish PE students compared to the Ukrainian sample. The study of Boyle et al. [63] showed differences in academic performance between Asian and non-Asian students from Australia. However, both groups attended the same university, so they shared the requirements and grading system. Conversely, the present result may depend on differences in grading systems or on a higher requirement for Ukrainian students. More research is needed to confirm and explain the present result.

This study showed that Polish PE students scored higher in emotional stability and extroversion as a personality trait than Ukrainian undergraduates. The Polish sample appears to present a personality pattern more similar to that of athletes [38,39,40]. Consistent with previous studies, differences in levels of particular personality traits were found across various countries, continents, and cultures [67,68,69]. In particular, extroversion (assertiveness) appears to be more sensitive to the cultural context [81]. Studies on the quality of life of Ukrainian children, adults, and seniors have shown consistently low levels of mental health (e.g., the impact of emotions on everyday activity, vitality, anxiety, and emotional fatigue) [82]. Ukrainian students showed significantly lower mental health, vitality, and emotional role functioning compared to students from Australia, England, China, Canada, and Sweden [83,84,85,86,87]. Pavlova [82] explained these differences by the lack of stability in Ukraine and the impossibility of planning for long-term goals. The mean trait profiles of cultures are currently explained by the evolutionary, ecological, and cultural theories of personality [67]. McCrae et al. [69] explained cultural differences from the perspective of individualism and collectivism, which affect many psychological processes. However, recent research shows that evolutionary theory may be more useful in explaining global variation in human personality [88].

Contrary to expectation, Polish and Ukrainian samples do not differ in academic motivation. Previous research showed that goal orientation differed in Asian and non-Asian cultures [63,66]. The differences were explained using the individualistic vs. collectivistic (independent vs. interdependent) approach. Asian cultures present a greater socially based collectivistic orientation, which determines performance goal orientation, whereas Western cultures are more individualistically oriented, which determines mastery goal orientation [63]. However, both Polish and Ukrainian countries share Western culture, which may explain similar motivation levels among undergraduates in this study. A meta-analytic review did not find cultural differences in motivation between countries classified as belonging to one of the following three continents: Asia, Europe, or North America [26]. Ryan and Deci [22] argue that functional autonomy is universal and relies on basic and universal needs, so human motivation, engagement, and learning should not differ across cultures. However, autonomy and controlling behavior, in addition to cultural and ethical norms, may distinguish various countries and be perceived more or less negatively, dependent on the culture. Although Poland and Ukraine are Slavic countries with a common border, they differ in terms of political system, standard of living (Ukraine is a low-income country), and socio-moral customs. The differences between these countries concern the social role and prestige of the teacher. In Poland, the teaching profession has a relatively low social rank due to its low economic status, particularly concerning workload. In Ukraine, obtaining teaching qualifications guarantees work and economic stability and is associated with high prestige and social respect, especially in small towns and villages. In both countries, the choice of field of study is consistent with sports interests and passion for exercise, which can explain the same level of autonomic motivation of PE students. Athletes usually work as coaches at the end of their sports careers.

### 4.3. Association of Academic Achievement with Motivation and Personality

This study found a positive relationship in academic performance with the intrinsic motivation scale and its subscale to know in all research samples, except males. The subscale of intrinsic motivation to accomplish correlated positively with GPA in the total sample of PE students and in both Ukrainian and female groups. In addition, a positive association was found between academic achievement and intrinsic motivation to experience stimulation in the Polish sample. Academic performance was unrelated in this study to extrinsic motivation and amotivation. Among the three scales of academic motivation (IM, EM, and AM), only intrinsic motivation was a predictor variable for academic performance. Although some differences regarding gender and country were presented in the relationship between GPA and motivation, the results of this study are consistent with previous research, showing that intrinsic motivation is a predictor of academic achievement [22,24,25,26,29,43,44,45,46,47,48]. However, intrinsic motivation explains only 2% of the variance in GPA, less than that explained in a previous study [29].

Other variables, such as gender, country, and personality traits, showed more reliable connections with GPA. Both demographic variables, gender and country, collectively explained 8% of the variance in academic performance. Furthermore, all five personality traits explained a further 8% of the variance in GPA. Although each of the five personality traits correlated with GPA in various configurations depending on gender or country, intellect and conscientiousness (with the exception of the female sample) consistently correlated with GPA. The two traits were also found to be significant predictors of GPA. Conscientiousness plays a key role as a predictor of achievement motivation, which was previously evidenced in numerous studies [29,30,31,32,33,34,35,36,37,43,44,45,46,47,48]. Conscientious people are self-disciplined, reliable, ordered, dutiful, hard-working, well organized, and systematic [27]. They tend to deliberate and transcend themselves while achieving higher performance in school and work or in developing interests, hobbies, and passions. Extreme conscientiousness may be associated with a higher risk of excessive perfectionism and workaholism. Conscientiousness facet-level traits of activity and self-discipline were found as predictors of exercise behavior and better sports performance [38,89,90,91]. Raynor and Levine [90] examined associations between the five-factor model of personality and health behaviors among American college students. Highly conscientious students were more likely to engage in exercise and other health-related behaviors. Results of research into the relationship between personality and self-determination of exercise behavior suggests that conscientious individuals exercise because this behavior can satisfy the need for competence [91].

Intellect (openness) was, however, also found to be positively correlated with tertiary academic performance in a systematic review and meta-analysis [36]. Lounsbury et al. [92] showed that openness was positively related to final grades in an undergraduate psychology course, with high scorers using learning strategies that emphasized critical thinking. There is some evidence that intellect as a personality trait was associated with general intelligence, as well as with both verbal and nonverbal intelligence [93]. Openness (intellect) is related to a rich imagination and preference to fantasize, creative thinking and achievement, aesthetic sensitivity, various hobbies, openness to experiences, and inner feelings [27]. Learning of knowledge and new ideas is related to cognitive curiosity, so the association between openness (intellect) and intrinsic motivation and high academic achievement seems justifiable.

For the first time, the present study explored the underlying mechanism by which intrinsic motivation influences academic performance through intellect, in addition to via conscientiousness, in interaction with gender in PE students. Intrinsic motivation can affect academic success among students with higher levels of intellect and highly conscientious men. Previous research attempted to explain the variance of academic performance, examining the interaction between low intrinsic motivation and high conscientiousness in the moderation model [46,47,48]. Komarraju et al. [29] found that conscientiousness partially mediated the relationship between intrinsic motivation to accomplish and GPA. The current study, which included gender in a mediation model, acquired more comprehensive insights and a deeper understanding of the specific relationship between the three variables of conscientiousness, academic performance, and intrinsic motivation.

### 4.4. Limitations of the Study

Participants in the present study were undergraduates studying exclusively in physical education departments. Thus, the results cannot be generalized across a whole student population. Furthermore, the research was performed at one university in Poland and one in Ukraine. The specific resources, requirements of students, and level of learning may be related to students’ GPA. The research should be replicated with a more representative sample of undergraduates at various universities. It would also be valuable to compare Ukrainian PE students studying at Polish universities with Polish and Ukrainian PE students in their countries of origin. The cross-sectional design of this study does not allow us to identify a causal link between variables, so the results of the mediation analysis should be considered with some caution. Future studies should be performed using a longitudinal design to examine the causal relationships between GPA, personality, and motivation.

## 5. Conclusions

Gender and cross-cultural differences contribute to academic achievements as much as selected personality traits, in particular conscientiousness and intellect/openness. In general, women are more motivated regarding academic achievements than men. Results of the mediation analysis indicate that those students who present a high level of intrinsic motivation and who are also conscientious men, or are intellectually open regardless of gender, have higher academic achievements. The results of this study may be useful for PE academic teachers to help motivate their students, depending on gender differences.

A systematic review showed the importance of interventions based on motivational processes to maintain significant changes in physical activity and sport over time [94]. The motivation treatment intervention (attributional retraining) was found to be effective in enhancing the academic success of competitive student-athletes [95]. Athletes reported benefits of an attribution-based motivation treatment in terms of perceived course success, performance, and persistence in making the transition from high school to college. In addition, SDT-based interventions, focusing in particular on training autonomy-supportive behaviors in teachers, were found to be useful in increasing students’ engagement and learning [22,96,97,98]. Autonomous motivation to exercise should be promoted in student populations at each level of education. This kind of intervention may be aimed to foster students’ perceptions of choice, personal mastery, fun, and excitement from exercise [99].

In recent years, the effectiveness of many new approaches to PE have been examined. A systematic review and meta-analysis indicates that cooperative learning interventions could be a useful teaching strategy to improve physical education students’ intrinsic motivation [100]. Research showed that support for novelty can predict satisfaction of basic psychological needs in PE students and positively influence their intrinsic motivation to undertake PA [101]. Hernández-Andreo et al. [102] proved that the Sports Education model is optimal to improve autonomic motivation in PE students. In addition, the flipped classroom was found to be an effective methodology that may positively influence academic performance and motivation in PE university students [103]. However, recent research shows PE students’ resistance to switching from the traditional model to other models focused on understanding the game, such as the Teaching Game for Understanding (TGfU) [104]. Thus, means of encouraging PE students to use modern person-centered methods, focused on increasing intrinsic motivation during PE classes, should be the main concern. Improving professional competences, in terms of experiences, knowledge, and skills acquired during physical education teacher education (PETE), can have an essential influence on the future quality of PE [105]. Kusurkar et al. [106] suggest that motivational processes should be taken into account as a crucial factor in PE curriculum development in schools. The focus on increasing intrinsic motivation in students, in particular, by including adequate feedback and emotional and autonomy support, may powerfully influence the outcomes of curricula. To achieve this aim, health educators should develop a gender-specific, motivation-based curriculum in universities aimed at increasing enjoyment, interest, and passion for PA among future PE teachers [12].

## Figures and Tables

**Table 1:** Demographic characteristics of the sample.

Demographic	Total Sample *n* (%)		Poland *n* (%)		Ukraine *n* (%)	
Variable	Women	Men	Women	Men	Women	Men
Level of study						
Bachelor	127 (29.95)	215 (50.71)	47 (20.98)	95 (42.41)	80 (40.00)	120 (60.00)
Master degree	36 (8.49)	46 (10.85)	36 (16.07)	46 (20.54)	0 (0.00)	0 (0.00)
Year of the study						
First	91 (21.46)	129 (30.42)	40 (17.86)	62 (27.68)	51 (25.50)	67 (33.50)
Second	46 (10.85)	84 (19.81)	22 (9.82)	52 (23.21)	24 (12.00)	32 (16.00)
Third	26 (6.13)	48 (11.32)	21 (9.37)	27 (12.05)	5 (2.50)	21 (10.50)
Specialization						
Teacher	77 (18.16)	102 (24.06)	50 (22.32)	63 (28.13)	27 (13.50)	39 (19.50)
Instructor/coach	86 (20.28)	159 (37.50)	33 (14.73)	78 (34.82)	53 (26.50)	81 (40.50)

**Table 2:** Gender differences in grade point average (GPA), motivation, and personality traits in physical education (PE) students.

	Women		Men				
Variable	*M*	*SD*	*M*	*SD*	*t* (422)	*p*	*Cohen’s d*
GPA	4.11	0.44	3.93	0.47	3.89	0.000	0.39
Intrinsic Motivation	55.50	12.66	50.69	14.20	3.53	0.000	0.36
To Know	21.52	4.76	19.52	5.26	3.95	0.000	0.40
To Accomplish	18.42	4.68	16.57	5.29	3.65	0.000	0.37
To Experience	15.56	5.10	14.60	5.21	1.87	0.063	0.19
Extrinsic Motivation	60.71	13.03	56.55	14.83	2.94	0.003	0.30
External Regulation	22.57	4.70	20.57	5.65	3.78	0.000	0.38
Introjected	18.56	5.76	17.47	6.03	1.84	0.067	0.18
Identification	19.58	5.09	18.51	5.51	2.00	0.046	0.20
Amotivation	7.85	4.93	9.74	5.95	−3.39	0.001	0.39
Personality traits							
Emotional Stability	11.40	2.74	12.79	2.84	−4.96	0.000	0.50
Extraversion	13.84	3.15	14.11	3.21	−0.85	0.396	0.08
Intellect	14.66	2.42	15.02	2.54	−1.42	0.157	0.14
Agreeableness	15.52	2.45	14.56	2.34	4.03	0.000	0.40
Conscientiousness	14.48	3.12	13.84	3.07	2.08	0.038	0.21

**Table 3:** A comparison of GPA, motivation, and personality traits of PE students from Ukraine and Poland.

	Ukraine		Poland				
Variable	*M*	*SD*	*M*	*SD*	*t* (422)	*p*	*Cohen’s d*
GPA	3.91	0.54	4.09	0.37	−4.10	0.000	0.39
Intrinsic Motivation	52.77	15.02	52.33	12.67	0.33	0.744	0.03
To Know	20.41	5.41	20.18	4.93	0.45	0.652	0.04
To Accomplish	17.07	5.59	17.47	4.70	−0.80	0.426	0.08
To Experience	15.30	5.49	14.68	4.89	1.21	0.225	0.12
Extrinsic Motivation	59.02	16.27	57.37	12.25	1.18	0.237	0.11
External Regulation	21.49	5.96	21.21	4.83	0.52	0.600	0.05
Introjected	18.18	6.41	17.63	5.50	0.95	0.342	0.09
Identification	19.35	5.97	18.53	4.75	1.57	0.117	0.15
Amotivation	9.17	6.01	8.88	5.31	0.53	0.598	0.05
Personality traits							
Emotional Stability	11.79	2.76	12.67	2.92	−3.16	0.002	0.31
Extraversion	13.61	2.97	14.36	3.34	−2.44	0.015	0.24
Intellect	14.67	2.60	15.07	2.39	−1.68	0.094	0.16
Agreeableness	15.11	2.39	14.78	2.45	1.39	0.164	0.14
Conscientiousness	14.79	2.87	13.45	3.17	4.54	0.000	0.44

**Table 4:** Correlation of GPA with motivation and personality in PE students.

Variable	Total	Ukrainian	Polish	Female	Male
Intrinsic Motivation	0.15 **	0.14 *	0.18 **	0.17 *	0.09
To Know	0.16 ***	0.14 *	0.21 ***	0.17 *	0.11
To Accomplish	0.15 **	0.16 *	0.12	0.22 **	0.07
To Experience	0.09	0.08	0.13 *	0.07	0.07
Extrinsic Motivation	0.09	0.11	0.10	0.15	0.02
External Regulation	0.09	0.09	0.12	0.10	0.04
Introjected	0.06	0.11	0.02	0.14	0.00
Identification	0.08	0.10	0.11	0.13	0.03
Amotivation	−0.03	0.01	−0.09	−0.08	0.03
Personality traits					
Emotional Stability	−0.04	−0.15 *	0.03	−0.07	0.05
Extraversion	0.10 *	0.00	0.17 *	0.17 *	0.07
Intellect	0.22 **	0.17 *	0.26 ***	0.25 ***	0.23 ***
Agreeableness	0.05	0.02	0.13 *	0.07	−0.01
Conscientiousness	0.19 ***	0.31 ***	0.17 *	−0.02	0.28 ***

* *p* < 0.05, ** *p* < 0.01, *** *p* < 0.001.

**Table 5:** Hierarchical regression results for academic performance.

		95% *CI* for *b*					
Variable	*b*	*LL*	*UL*	*SE b*	β	*R* ^2^	Δ*R*^2^
Step 1						0.08 ***	0.08 ***
Constant	4.01 ***	3.93	4.10	0.04			
Gender	−0.18 ***	−0.27	−0.10	0.04	−0.19 ***		
Country	0.19 ***	0.10	0.27	0.04	0.20 ***		
Step 2						0.09 ***	0.02 *
Constant	3.67 ***	3.39	3.95	0.14			
Gender	−0.17 ***	−0.26	−0.08	0.05	−0.18 ***		
Country	0.19 ***	0.11	0.28	0.04	0.21 ***		
IM	0.01 *	0.00	0.01	0.00	0.16 *		
EM	0.00	0.00	0.00	0.00	0.00		
AM	0.01	0.00	0.02	0.00	0.08		
Step 3						0.17 ***	0.08 ***
Constant	2.99 ***	2.52	3.47	0.24			
Gender	−0.17 ***	−0.26	−0.08	0.05	−0.18 ***		
Country	0.22 ***	0.13	0.30	0.04	0.23 ***		
IM	0.00	0.00	0.01	0.00	0.07		
EM	0.00	0.00	0.00	0.00	0.02		
AM	0.01	0.00	0.01	0.00	0.07		
ES	−0.01	−0.03	0.01	0.01	−0.06		
E	0.01	−0.01	0.02	0.01	0.05		
I	0.03 ***	0.02	0.05	0.01	0.18 ***		
A	−0.01	−0.03	0.01	0.01	−0.05		
C	0.03 ***	0.02	0.04	0.01	0.20 ***		

**Note.** IM = intrinsic motivation, EM = extrinsic motivation, AM = amotivation, ES = emotional stability, E = extraversion, I = intellect, A = Agreeableness, C = Conscientiousness. Gender was coded: Women = 0, Men = 1; Country was coded: Ukraine = 0, Poland = 1. Statistical symbols are: *b* = unstandardized beta, *CI* = confidence interval, *LL* = lower level, *UL* = upper level, *SE b* = standard error of unstandardized beta, β = standardized beta, *R*^2^ = coefficient of determination, Δ*R*^2^ = change in *R*^2^ between two equations in a following steps of regression. * *p* < 0.05, *** *p* < 0.001.
